# The Physiology of the Circulation

**Published:** 1859-11

**Authors:** John C. Dalton

**Affiliations:** Professor of Physiology and Microscopic Anatomy


NEW YORK MONTHLY REVIEW,

AND

BUFFALO MEDICAL JOURNAL.

**VOL. 15.** NOVEMBER, 1859. **NO. 6.**

### ORIGINAL COMMUNICATIONS.

ART. I.—*The Physiology of the Circulation. A Course of Lectures delivered at the College of Physicians and Surgeons, New York, in the Fall Term of* 1859. By John C. Dalton, Jr., M. D., Prof. of Physiology and Microscopic Anatomy.

### LECTURE I.

(September 20, 1859.)

Subject of the Course.—Nature of Physiological Studies.—Relations with Anatomy.—Methods of Physiological Investigation.—Experiments on the Living Body.—Objections to this method.—Their Futility.—Difficulties in the way of Physiological Experiments.—Complication of Physiological Phenomena.—Their Variability.—Influence of External Agents.—Of Internal Conditions.—Means of avoiding Error from this Source.—Special Rules for conducting Physiological Experiments.—Variation of results in different Species of Animals.—Difference between Direct and Indirect Results of an Operation.—Examples.—Individual Peculiarities of different Animals.

Gentlemen :—

The Course of Lectures which we are now about to commence, is a Special Course on the Physiology of the Circulation ; and I think we shall find that in selecting what might perhaps be regarded as a somewhat limited subject, we do not in reality narrow the field of study which is opened to us, but, on the contrary, enlarge it, for all purposes of real improvement and experimental inquiry. For each special group of the animal functions is found, upon examination, to be vastly richer in its details, and more complicated in its relations, than we should anticipate from a mere cursory inspection ; and in confining our attention, for the present, to the phenomena of the Circulation, we shall gain space and time to follow out these phenomena in many important connections, and to study in detail the many varied conditions upon which they depend.

But before commencing the study of the particular facts relating to our present subject, it will be worth while to say a few words on the nature of physiological investigations in general, and on the manner in which they should be pursued. For physiology is a science of such recent growth, and is concerned with phenomena of so peculiar and special a character, that we cannot properly appreciate the value or significance of the facts presented to us, unless we fully understand the manner in which they are discovered, and are familiar with the tests to which they should be subjected.

Physiology, gentlemen, follows in the natural order of study upon Anatomy, with which it always is, and must be, most intimately associated. No one can examine the situation, size, and shape of an internal organ, without asking himself, at least, what are its functions, what purpose does it serve in the animal economy, and what are its relations to the neighboring parts. Anatomical facts, by themselves, would be destitute of interest, unless they were connected in some way with physiological actions. On the other hand, it is evident that we cannot advance a single step in physiology without the aid of anatomical facts to rest upon. Not only must we know the locality, form, and texture of the principal organ before we can study its actions, but there are a thousand anatomical details, which are requisite to enable us to perform our experiments, or even to make the commonest physiological observation, without danger of misinterpreting its results. How essential is it, for one who is investigating the physiology of the chyle and lymph, to know all the divisions and inosculations, both constant and occasional, which exist between the different parts of the lymphatic system, or between the lymphatics and the veins. Such an anatomical relation, if unknown or neglected, might entirely vitiate the result of an otherwise well contrived and faithfully executed experiment. The experiment of tying the pancreatic duct, for example, was supposed to prove the secretion of this gland to be unnecessary for the digestion of fatty substances in the intestine, until it was discovered that the pancreas has habitually two ducts instead of one, and that its secretion may therefore find its way into the intestine by the second of these ducts, after the first has been obliterated. The knowledge of anatomical details is accordingly essential to the physiologist. They are the necessary tools, without which he cannot do his work thoroughly, nor engage safely in any experimental investigation.

It is natural, therefore, that the study of anatomy should have preceded, in the order of time, that of physiology. The former was already far advanced, as a science, before the latter had begun to be successfully cultivated; and the study of the one seems naturally to open the door for that of the other. It is on this account that the connection between the two has sometimes been supposed to be more intimate than it is in reality. Anatomy is sometimes regarded, not only as a necessary assistance to the study of physiology, but as actually furnishing the data from which physiological truths may be derived, as consequences or corollaries; particularly when the study of microscopic anatomy first began to be successfully cultivated, it was thought that such thorough and detailed acquaintance with the minute structure of the organs, would necessarily give us a more complete insight into their functions. Every variety of physiological action was regarded as necessarily connected with a special arrangement of the anatomical elements; and it was expected that the peculiar function of an organ or tissue would easily be detected, as soon as we had mastered all the details of its anatomical structure, and had learned the exact distribution of its fibres, its cells, its blood-vessels, and its nerves.

It is necessary, however, that we should understand in the outset, if we are to pursue with success any department of physiology, that we cannot rely upon such methods of investigation. There is no such connection between the structure of an organ and its function, as will enable us to deduce the one from the other. However minute the structure, and however simple the function, the latter is always to be ascertained by separate and direct experiment, and in no other way. A knowledge of structure therefore, enables us to perform the experiment, but not to foresee its result. The connection between the structure and function, when this connection exists, is usually of such a nature that we cannot understand it, even after we know both the anatomy and physiology of an organ; much less anticipate it beforehand. The physiological action of a part is accordingly, in most instances, a vital property, the existence of which cannot be explained from anatomical facts, but can only be established and demonstrated by direct experiment.

Here, for example, is one of the principal cranial nerves (third division of the Fifth Pair), which has been dissected so as to exhibit the course of its different branches. The nerve emerges, as you see, from the foramen ovale at the base of the skull; and one of its branches, after supplying the tongue and the parotid gland, passes forward, through the dental canal, in the inferior maxilla, to the region of the chin and lower lip; while the other breaks up into ramifications which are distributed to the muscles moving the lower jaw. Now, there is no difference in the external aspect of these two branches, and if we examine them by the microscope, we shall find them both composed of nervous filaments having the same size, the same structure, the same arrangement. To all appearance the anatomical constitution of the two branches is the same; and yet their physiological properties are totally distinct, for experiment shows one of them to be motor, and the other sensitive.

We cannot even rely upon the ultimate distribution of the nerves, in order to decide whether they are sensitive or motor, since the muscles also receive sensitive filaments to which they owe their sensibility, as well as motor filaments which give them the property of contraction. The superior laryngeal nerve for example, sends a branch which is distributed to the arytenoideus muscle; but this branch is nevertheless sensitive and not motor, for experiment shows that its division neither paralyzes the muscle to which it passes, nor does its excitement by galvanism produce any convulsive action in the same part.

Here are two glandular organs, the parotid and the pancreas. Both have the same firm consistency, whitish color, and lobulated texture. They are both composed of similar rounded follicles, lined with glandular cells, and opening into tubular excretory ducts. They are so much alike that, among the Germans, the pancreas has been named after the parotid, and called the "abdominal salivary gland" (*Bauch-speichel-drüse*). Both of these organs, furthermore, are supplied with the same arterial blood; and yet one of them secretes saliva, a thin, watery fluid, containing less than two parts per thousand of organic matter; while the other produces the pancreatic juice, a viscid secretion, containing ninety parts per thousand of organic matter, and quite different in its physiological properties from any other glandular fluid.

Even where a difference in anatomical structure exists between two organs, there is often no necessary connection to be detected between this difference of structure and the peculiarities of their physiological action. The voluntary muscles, for example, are composed of large, cylindrical, striated muscular fibres, the contraction of which is sudden, easily excited, and instantaneous in duration; while the involuntary muscular fibres are small, flattened, fusiform and ribbon-shaped, and, at the same time, sluggish in contraction. But a knowledge of the anatomical characters of these two fibres would never be sufficient to teach us the nature of their physiological peculiarities. The difference between them can only be learned from experiment and observation.

We find, therefore, that anatomy is subservient to physiology only as a means of study. Physiology as a practical pursuit, rests upon a different basis, and is to be carried on by other methods, since it leads our investigations into another domain, and carries them far beyond the limits which confine the labors of the anatomist. He is interested, as an anatomist, only in the size, the location, the form, and the structure of the different parts of the body. He enumerates the processes and foramina of the bones, the attachment of tendons and ligaments, and the distribution of blood-vessels and nerves. These details are, all of them, important, and possess a certain amount of scientific, as well as practical, interest; but they can never, of themselves, satisfy him who has once commenced the study of the animal frame, whatever may have been his primary object. The vital activity of these different parts, and their physiological relations with each other, soon become infinitely more important, in a scientific point of view, than the mere particulars of form and structure. What should we think of a naturalist whose whole interest was absorbed in the dry bones and the stuffed specimens of a zoological museum, but who could give no account of the history, action, and habits of the animals during life? Such is the position of the student who has mastered the details of anatomy, but has not used this knoweledge as a means of introduction to physiology.

The naturalist who comprehends his subject, goes much further than this. The great Audubon, in preparing his masterly work on the Birds of America, did not pursue his labors alone in the library and the dissecting room. He penetrated into the forests and the mountains, and, concealed in swamps and thickets, lay patiently in wait to watch the movements of the birds, and to study their peculiar habits and their modes of life.

We then, gentlemen, as physiologists, are the naturalists of the living body. Our object is to study its movements, to learn its habits and functions. Our business is to penetrate into the recesses of the internal organs, and to lie in wait, so to speak, to watch their complicated processes, and to learn the secrets of their physiological action. For the anatomist, the body is to be examined as an inert and lifeless frame, composed of bones, muscles, ligaments, and membranes, with the fluids contained in their appropriate cavities and tubes. But for us, it is an animated and active organism, with the muscles alternately contracting and relaxing, the lungs inhaling and exhaling the atmosphere, the heart beating, the fluids circulating incessantly from one extremity to the other, and every fibre palpitating with life and sensibility. Such is the field presented to the study of the physiologist. It is easy to understand that this is a very different subject, both in its nature and its mode of study, from that which engages the attention of the anatomist. It is the difference between life and death, between force and inertia, between repose and activity.

From what I have just said, we can readily comprehend what are the appropriate methods for pursuing the study of physiology. It is only by experiment and observation upon the living body, that we can learn what are the actions and processes which go on in it during life. Simple observation, indeed, will teach us but little. Most of the organs are concealed in the interior of the body; and we must, accordingly, resort to experiment in order to discover their functions. Even for the most palpable and external of the vital phenomena, as, for example, the inspiration and exhalation of gaseous matters by the breath, some artificial contrivance is usually necessary in order to secure accuracy in the results of observation. It is, therefore, by experiment upon the animal frame and its immediate products that we mainly succeed in the acquisition of physiological knowledge.

It is true that there are many other modes of experiment which will assist us more or less in our study of the vital actions. Certain physical and chemical investigations are often of service, by leading us into the right path, and by indicating the direction in which our purely physiological examinations are to be pursued. Analogical surmises, and inferences from what we know of other phenomena, may also aid us in reaching the solution of the doubtful question which is to be decided. But in all these cases the physical or chemical knowledge which we obtain, or the scientific analogies which may be presented to us, can only be useful so far as they may suggest probabilities, and thus direct our researches in the proper channel. In every instance the decisive experiment must be one which is concerned only with the animal organism. We may experiment, for example, upon endosmosis and exosmosis, with cups and bladders in the laboratory, and may gain, in this way, a certain amount of knowledge as to the nature and operation of these forces; but it is impossible to ascertain the manner in which they actually take place in the interior of the body, or to measure the processes of exudation and absorption of the animal fluids in the course of the circulation, except by experiment upon the living body itself.

If we were to depend upon any other mode of investigation than that which I have just mentioned, for the definite solution of physiological questions, we should be constantly liable to erroneous conclusions. In all preliminary investigations it is our object to make the conditions of the experiment as similar as possible to those which exist in nature; and the more nearly these conditions resemble each other, the more valuable will be the result. But they can never be made absolutely identical; and the result will, accordingly, be more or less doubtful so long as the conditions of the experiment are in any degree artificial.

For it must not be forgotten that the object of all our researches is to become acquainted with the physiological phenomena, *as they actually take place in the living body;* and direct examination shows that these phenomena, even when of a physical or chemical nature, are often quite different from what we should expect from our general knowledge of chemistry and physics, or from the results of previous physical and chemical experiments.

Here, for example, is a thin solution of hydrated starch, into which I pour a few drops of iodine water. The mixture, you observe, instantly assumes a deep, opaque, blue color, which is characteristic of the reaction of these two substances. Here, on the other hand, is a small quantity of an animal fluid, viz., freshly secreted saliva. If we now, instead of mingling the starch and iodine by themselves, mix them in the test tube containing the saliva, no reaction takes place between them, and no blue color is produced. We can even, as you see, destroy the blue color already produced by the mixture of starch and iodine, by adding to it an equal quantity of saliva.

The chemical reaction, therefore, does not take place in the presence of the animal fluid, as it would do under ordinary circumstances.

Here is a little distilled water, to which I add a drop or two of honey. I then put into the solution a little sulphate of copper, and afterwards some caustic potass, both dissolved in water. The mixture now takes a clear blue tinge, owing to the presence of the sulphate of copper. On boiling the mixture, it rapidly becomes turbid and yellow, and is soon filled with an abundant, bright, yellowish red deposit of the sub-oxide of copper. This is Trommer's test for grape sugar, and is one of the most convenient and certain means of detecting that substance when in a state of purity.

Here, however, is some gastric juice from the dog. I put into it a drop or two of honey, and thoroughly mix the two fluids together. I then add sulphate of copper and caustic potass, for Trommer's test, as before. The mixture, you see, instead of a blue color, has now a bright purple or violet tinge; and on boiling it, though it loses the purple color and becomes yellowish, no deposit is thrown down, and the fluid remains simply opalescent. In these cases, the usual action of the chemical tests has been altered, on account of a peculiar organic matter existing in the animal fluids, which has its own special properties and reactions, and interferes, accordingly, with the action of other substances which may be present.

It is for a similar reason that the action of drugs upon the living body can never be anticipated with certainty from a knowledge of their ordinary chemical properties; and therapeutical systems based upon such purely chemical data, are usually found, in practice, to be erroneous, because some unforeseen peculiarity of action almost always intervenes, in the interior of the body, to disturb our calculations. Accordingly, we can seldom determine what changes a drug will undergo on being introduced into the system, except by actually searching for it in the blood and secretions. If iodine, for example, be taken internally, it is discharged from the body with the secreted and excreted fluids,—saliva, perspiration, urine,—but never in a free state. It always enters into an intimate organic combination in the animal fluids, and accordingly cannot be detected, either in the blood or the secretions, by the test of starch, unless the organic matter with which it is in union be first destroyed by the addition of an acid. The precaution, therefore, which has been sometimes inculcated, of forbidding to patients the use of bread, or other starchy food, for an hour or two after taking iodine, is entirely unnecessary; for we find that iodine will not combine with starch in presence of an animal fluid, but unites, in preference, with the organic matter which is in solution.

The malates, citrates, and tartrates, again, of soda and potass, when taken into the system, do not appear under their own form in the secretions; but the organic acids of these salts are replaced, during their passage through the circulation, by carbonic acid, and they are, accordingly, discharged, in the excreted fluids, as the carbonates of the same bases.

It is by experimenting, therefore, upon the living body that we shall endeavor to gain an insight into its physiological operations. That is the only source from which we can derive absolute and positive information, and it is to that test that all unsettled physiological doctrines must finally be submitted.

I wish, however, for the time which remains to us in this lecture, to examine, in the first place, certain objections which have been made against experimenting upon the living animal; and, secondly, to enumerate the more important difficulties which lie in the way of this mode of investigation, and the precautions which are necessary to insure its ultimate success.

And, first, as to the objections urged against it.

I would say, however, to begin with, that whatever these objections may be, it is certain that they cannot destroy the comparative value of this mode of study; since, however deficient a method this one may turn out to be, it is evident that *there is no other upon which we can rely.* This follows from what we have already said as to the nature of the vital functions, and the manner in which they are naturally performed. There is no alternative left us, therefore, if we wish to study physiology, of choosing this course or another, for this is the only mode by which we can hope to obtain the desired information. There is no other way of learning the operation of physiological phenomena than by seeing how they actually take place in the organism itself. It is only in the liver that we can investigate the formation of the bile, and only in the intestine that we can follow the changes of its decomposition; and we might as well try to find the Northwest Passage by exploring the Southern Ocean, as to study the phenomena of the circulation *anywhere* but in the blood-vessels of the living body.

The objections to this method have originated, however, as I think, for the most part, in an undue estimate of other means of study, rather than in positive experience of its own want of value. Experimental physiology is, by some, underrated and neglected—not because it has been tested and found wanting, but because it is easier to construct a "physiology of probabilities" out of such materials as Analogy, and what is called the "Unity of Scientific Truth." That, however, is not the kind of physiology which we propose to study; and, accordingly, these methods of inquiry are not the ones adapted for our employment.

The principal difficulty which has been directly urged in regard to the method of experimentation upon the living body is, that there is danger of fallacy in such investigations. The objection has been expressed as follows: "Nature tortured," it is said, "does not tell the truth by any means as clearly as nature with its ordinary relations." It is not meant by this that the pain which the animals suffer, while being experimented on, produces false appearances, and so deceives the experimenter. It is very rarely necessary to inflict pain for purposes of physiological experiment. The employment of ether and chloroform, which are fully as useful to the physiologist as to the surgeon, enables us to do the requisite preliminary operations without the animal's experiencing any sense of suffering, and even without his consciousness. It is only certain experiments upon the nervous system which cannot be done without inflicting pain upon the animal; but in these instances the existence, or otherwise, of a painful sensation is the special object of the experimenter's research; and its indications, accordingly, so far from being liable to mislead him or to vitiate his results, are the very means upon which he depends to arrive at a correct conclusion.

The real meaning of the above-mentioned objection, as it is usually understood, is that the necessary steps of the preliminary operation, the incisions, ligatures, mutilations, &c., interfere with the usual action of the animal organs, and introduce a disturbing influence into the play of the vital functions; and that nature is thereby "tortured," or distorted, to such an extent that the experimenter is liable to meet with false and uncertain phenomena, and to derive from them erroneous conclusions.

This objection, however, is one which certainly would never be made by a practical experimenter, for the reason that the difficulty to which it refers is always present to his mind, and is one against which he is constantly guarding by unremitting precautions. This, in fact, is the very first and the simplest lesson which is learned by the experimental physiologist. You might as well object to the researches of an astronomer, that the refractive power of the atmosphere distorts the rays of light passing through it, and thus falsifies the appearances presented by the telescope, and vitiates the conclusions of the observer. The astronomer is perfectly familiar with this refractive power of the atmosphere. He knows the mode of its operation, and makes allowance for it in all his calculations. So the physiological experimenter, in prosecuting his observations on the living body, sees at once that the external agents which he employs in his operations, and the manipulations to which he is compelled to resort, themselves exert a certain amount of influence in producing the final result. It is always his object, in contriving an experiment, to reduce these disturbing influences to the smallest possible compass and the simplest possible form. It is frequently necessary to vary the method of procedure, for the same experiment, in order to determine how much, if any, of the result finally produced is attributable to the principal conditions, and how much to the accessory manipulations. Abundant instances of this are to be found in the history of investigation upon almost every physiological question; and it would be a bungling experimenter, indeed, who left out of consideration the physical conditions which he himself had employed in operating upon the living body.

I have already stated, gentlemen, that we cannot depend, in studying physiology, upon the examination of external phenomena, and that we must finally resort to experiment upon the animal organism itself; and I also mentioned that the principal reason for this necessity was that we could never reproduce externally the exact natural conditions which are present in the living body. It follows, therefore, that our first care, in experimenting, should be to alter these natural conditions as little as possible, in order that the physiological phenomena themselves may be undisturbed. Is one of the internal organs to be reached and examined by the experimenter? The opening in the abdominal walls should be of small extent, and made without exposing or injuring the neighboring parts. Is a specimen of blood to be withdrawn from a particular vein, for purposes of comparative analysis? The vein upon which we are to operate should be exposed carefully and rapidly, the ligatures applied before the natural course of the circulation is disturbed, the blood withdrawn just as it comes fresh from the capillary vessels, and collected in such a way as not to allow of the admixture of any other of the circulating fluids. Of course, the conditions of our experiment can never be precisely those which exist in the ordinary state. It is because the vital operations of the body are naturally concealed from observation that we are obliged to resort to experimental contrivances in order to gain some knowledge in regard to them. But with proper care and judgment in their employment, these contrivances need not interfere with the principal phenomenon which is to be observed. Our experiments can never, it is true, present us with a complete panorama of the animal functions in the uninterrupted harmony of their actual operation; but they may enable us, by separate observations and the study of isolated phenomena, to comprehend the mode in which all the organs act together, in their natural and undisturbed condition.

There are, however, other and more serious difficulties in the way of physiological investigation, and certain precautions which are indispensable for the practical experimenter, beside those already mentioned.

I. In the first place, the principal peculiarity which distinguishes the phenomena of organic life is that they are complicated. The result of a physiological experiment is often not a direct and simple one, as in the case of physical or chemical investigations, but depends upon the combined operation of various forces, each of which exerts its own share of influence. The same phenomenon, therefore, is susceptible of different interpretations, until we have finally mastered all the elements which are concerned in its production. If the pneumogastric nerve be divided, for example, in the dog, in the middle of the neck, respiration soon becomes diminished in rapidity. At the end of a few days the animal dies, and the lungs are found in a peculiar state of engorgement and solidification, impermeable to the air, and evidently incapable of performing their functions. Coagula are also frequently found in the cavities of the heart and the pulmonary vessels, sufficient to interfere with the natural course of the circulation. But we should be mistaken if we supposed that the division of the nervous filaments distributed to the lungs was the direct cause, either of the engorgement of the pulmonary tissues, or of the coagulation of the blood in the pulmonary vessels. For the pneumogastric nerve is also distributed to various other organs, and the interruption of its influence over them disturbs indirectly the functions and nutrition of the lungs. The muscles of the larynx, in particular, are supplied by the recurrent laryngeal nerve, a branch which leaves the pneumogastric at the lower part of the neck. A paralysis of these muscles stops the regular movements of the glottis, by which it usually expands at the time of inspiration and collapses during expiration, and the air accordingly no longer finds a free access to the trachea and lungs; the pulmonary circulation consequently becomes sluggish, the blood coagulates in the vessels, and the pulmonary tissue becomes engorged and solidified. The substance of the lungs, therefore, is altered, not because its own nerve has been cut off, but because the branch distributed to the larynx has been divided at the same time. The proof of this is, that if the recurrent laryngeal nerve be divided on both sides without injuring the main trunk of the pneumogastric, the lungs after a time present the same kind of engorgement and solidification as in the former instance.

If a dog be fed with a mixture of meat and boiled starch, we know that the starch is rapidly converted into sugar; for the sugar may be found, if sought for at the proper time, in the duodenum of the animal, where the intestinal fluids act upon the food, and transform its starchy ingredients. But if the examination be delayed a little too long, no sugar will be discovered; because, simultaneously with the digestion of the food, the process of absorption is going on in the intestine, and the saccharine material which is produced is rapidly removed and carried into the circulation. There are many instances in which, by these compound processes of secretion and absorption, of production and decomposition, effects are produced which could not be obtained from either of them alone.

II. In the second place, the vital phenomena are variable at different times. The living body is not an inert structure composed of unchangeable materials. It is an active and impressible organization, susceptible to the influence of external causes, and subject even to variations of an internal and periodical nature. It is on this account that the physiologist, in commencing his practical investigations, finds it so difficult to foresee the result of an experiment from one trial to another. An experiment which succeeds to-day will fail to-morrow, though repeated with the same care and observed with the same fidelity. This is because some unforeseen circumstance has arisen, which, though apparently too trivial to attract the attention of the observer, is yet sufficient to influence the animal organism or its products, and so alter the phenomena which they present.

I need not impress it on you, therefore, that in physiological experiments, more than in any other, it is of the first importance that the surrounding conditions should be always absolutely identical. The quantity of material used, the quality of the instruments, the time and order in which the different steps of the operation are performed, must all be arranged with precision; for any of these circumstances are liable to influence the result. I have here a bottle of venous blood from the ox. I pour some of it into another empty bottle; and, on shaking it up with atmospheric air, you see the dark blood immediately assume a bright vermilion tinge, just as it does in the capillaries of the lungs. Here is another bottle of venous blood, of similar appearance, from the same animal. On shaking it up with air as before, the color, you see, suffers but little perceptible change, but remains nearly as dark and venous as before; and yet these two specimens of blood were both taken from the same animal, drawn in the same way, and treated with the same agents. The only difference is, that the second specimen has been withdrawn from the vessels from thirty-six to forty-eight hours longer than the first. During this time a change has taken place in its organic ingredients which renders it less susceptible of reddening under the influence of the atmospheric oxygen.

Even where the animal fluid employed is perfectly fresh, a similar variation may be observed in its properties in two different experiments; because the vital condition of the whole body, or of some particular organ, is different on the second occasion from what it was on the first.

A striking example of this is to be seen in the secretion of the saliva, and its power of converting starch into sugar. All physiologists are now agreed that the saliva taken from the cavity of the mouth is capable of converting boiled starch into sugar; and yet the intensity of this action varies in a manner which has been hitherto altogether unexplained. At one time it requires ten or fifteen minutes to show traces of sugar in the mixture of starch and saliva, and at another, the sugar can be detected readily, by the ordinary chemical tests, at the end of half a minute. This is because some organic ingredient of the saliva, essential to its action on starch, is more readily secreted at some times than at others; but we have not yet succeeded in ascertaining why or when the variation takes place. Practically, in preparing for an experiment of this nature, we must always test beforehand the particular specimen of saliva which we are to use; for, otherwise, we can never be sure that its activity is the same with that which was secreted a day, or even a few hours, before.

But there are many instances in which we know upon what particular circumstances such variations in the vital phenomena are dependent. They are frequently owing to a difference in the size, age, sex, vigor, constitution, &c., of the animals employed for experiment. Age alone will sometimes have such an effect as to alter entirely the effect of certain injuries or operations. I have already mentioned that if the pneumogastric nerves be divided in an adult dog, the respiration becomes diminished in rapidity, and death comes on gradually at the end of from three to six days. But if the same operation be done upon pups but a few days old, a violent choking at once occurs, owing to a more complete closure of the glottis; and death supervenes very rapidly. Legallois has even seen a young dog, under these circumstances, die instantaneously from suffocation,—an accident which never happens with the adult animal, since, in him, the orifice of the glottis is only diminished in size by paralysis of the laryngeal muscles, and not completely obstructed.

Among the most important of these variations are those which depend upon periodical changes in the vital functions, recurring, perhaps, at intervals of a day, or even of a few hours. An experiment may have different results in two animals, precisely similar in their external appearance, because their internal condition is not the same in reference to the alternations of waking and rest, of fasting and digestion. Here are two specimens of blood, both drawn yesterday from the vessels of the dog. In both coagulation has taken place, and in both the clot and serum have separated from each other. But in one, you see, the serum is of a clear amber color, perfectly homogeneous throughout. In the other, the surface of the serum is covered with a white, milky film, which is composed of minute fatty granules and molecules in a state of emulsion. This peculiar appearance is due to the blood having been drawn during the digestion of food containing fatty ingredients. A few hours later it would have entirely disappeared, and the two specimens of blood would then be alike.

Enough has been said, therefore, to show why physiological experiment has been sometimes thought subject to deception, and unreliable, and to show, also, how erroneous in reality such a conclusion would be. The vital phenomena of the living body, in point of fact, are not in themselves variable, any more than other natural phenomena; they are only dependent on a greater variety of surrounding conditions. The result of a physiological experiment will always be the same, provided the conditions under which it is tried be also identical. If, therefore, we find that an experiment which succeeded yesterday fails to-day, or has a different result, we shall always discover, on examination, that there is some difference in the mode of its performance, which causes the variation. There are no contradictions in nature, but only in our imperfect observation or interpretation of the natural phenomena. So far from being confused or disappointed, therefore, by such an apparent contradiction, we should simply set ourselves at work to discover the cause upon which it depends; and when this is accomplished, the certainty and extent of our knowledge will be increased, instead of diminished.

III. It follows from what has been said, in the third place, that the apparent contradictions of physiological experiment, instead of destroying the value of its results, may in reality serve to enhance the accuracy of our knowledge, and to open to the inquirer new paths of investigation. It is, accordingly, a most injudicious thing for the experimenter to neglect or throw aside any phenomena which he may notice, because they appear to be in opposition with others which he has already observed. The apparent opposition between the two is of itself an important fact, which may turn out, if properly investigated, to be fruitful in new discoveries. Suppose, then, that we have observed certain results to take place in a series of experiments, and have deduced from them certain conclusions. A year afterward, on repeating the trial, we meet with results which are not in accordance with the previous ones. Are we, in this case, to neglect the phenomena which we have observed last, and adhere to our old opinion? Or are we to suppose that there was some mistake about the former experiment, and continue to reason from our present results as if we had never met with any other? There are persons who would adopt one or the other of these alternatives, according to the character of their mental constitution, or the nature of the facts in dispute. But either method would be wrong, for they are both based on a mistaken principle. To neglect a fact actually observed, is as much a falsehood in science as to imagine one that has never been seen. Neither the new nor the old observation accordingly is to be disregarded, but equal value should be given to both. The one does not invalidate the other; and we know that further investigation will certainly reconcile the apparent discrepancy.

I need not remark, however, that in order to depend thus implicitly upon his experiments, the physiologist must be habituated to observe every phenomenon, precisely as it occurs, without reference to anything else than the simple result of the experiment. Without such precision in the observation of details, the experiment will often be worthless; and particularly if the experimenter be unduly influenced by the supposed explanation which he desires to give of the phenomena in question, he may allow certain appearances to escape his notice or his memory, which are essential parts of the entire experiment. Subsequently, then, he may be puzzled by meeting with contrary appearances, when, in reality, the results of both experiments are essentially the same, and would not even appear to be different if he had not neglected or forgotten some of the details in the former instance.

In experimenting with animals, accordingly, we must first keep in mind the complicated nature of the phenomena which they present. We must remember, especially, that the *result* of an experiment is one thing, and our *interpretation* of it another. Our interpretations may afterwards turn out to be erroneous, or require to be modified, but the physiological phenomena are the same, and are not affected in any way by the alteration in our mode of explaining them. When our theories, accordingly, are changed with the progress of time, we do not lose anything in giving up the old ones for the new, because we do not sacrifice any one fact, however insignificant, which has ever been observed. We only change the hypothetical arrangement in which we had placed these facts, and enlarge it so as to include others equally valuable.

The physiologist, therefore, must never allow himself to regard these theoretical explanations as of more value than the actual phenomena which he sees to take place in the course of experiment. Whenever we observe any natural events or appearances, the constitution of our minds leads us at once to endeavor to reduce these appearances to some regular arrangement or order, and so subject them to the expression of some general law. But we must never regard these hypothetical laws as equal in importance to the phenomena which present themselves in nature. Any fact or event, actually observed, is superior in value to any theory or law ever framed in scientific language; for these latter are altogether temporary and ideal in their existence, while the phenomena to which they relate are positive, absolute, and imperishable. No doubt there are in nature certain laws or principles which are invariable, and to which the operation of all phenomena is subjected; but our only hope of learning what these laws really are, is in questioning nature herself by experiment, and relying implicitly on the answers which we receive. For the phenomena actually witnessed are the only real expression of the laws which regulate them; and any law which is not in accordance with every phenomenon that may be presented, is, *ipso facto*, false, and does not represent the real principle which exists in nature, and which it is our object to discover.

In practice, then, we must be ready to receive every appearance that may be presented during an experiment as equally absolute and valuable whether it corresponds with our previous ideas, or whether it leads us in a new direction and suggests the adoption of a more comprehensive theory. It is only when the mind of the observer has arrived at this condition that he is secure from self-deception, and that he is always certain of making progress, whatever may be the appearances which he meets with.

IV. Fourthly, there are certain rules, also, of a special nature, very important for the practical physiologist. One of these is that the same animal should never be used for more than a single experiment, nor the same experiment employed to demonstrate more than a single point. The principal reason for this is evident; for if the animal have already been subjected to an operation which disturbs the action of a single organ, we cannot depend upon the action of others, which may be affected by sympathy with the first. Every experiment, to be reliable, must be tried upon an animal in the uniform and standard condition of perfect health. If the pneumogastric nerves of a dog have been divided, we cannot safely make use of him for an experiment upon the saliva; for though there is no direct connection between this nerve and the parotid gland, the secretion may suffer from the indirect influence of the heart or the stomach. But beside this, there is another reason for the above rule, which relates more to the experimenter than to the animal upon which he operates. It is this. In an experiment, the mind of the observer always is, or should be, intent upon a single point. He wishes to ascertain positively and distinctly the action of a bodily organ under certain conditions, and he makes all his arrangements to secure accuracy and success in observing its details. Now, if he endeavors, at the same time, to use this opportunity for noticing other phenomena of a different nature, and disconnected with the first, he will find that his mind is not in a condition to appreciate both sets of appearances in a proper manner. Either, in taking care not to neglect the first part of the experiment, he will observe the second loosely and carelessly; or, in paying attention to both, he will find that neither of them have been conducted with sufficient accuracy to make them reliable. Besides, in most experiments upon living animals, a certain order and celerity in the mechanical manipulations are requisite to insure success; and if we try, at the same time, to introduce other contrivances, however simple, the chances are very great that they will interfere, just at the wrong moment, with some necessary part of the principal operation, and all the labor of the experimenter will be thrown away. Each experiment, then, should be contrived and executed by itself. This seems to involve a great expense of time and material. But if we try to economize by making one operation answer for two experiments, or by making one experiment settle two questions, we shall find that, instead of saving, we have lost. "One thing at a time" is nowhere a more necessary rule than in physiological experiment; for in that, to do one thing only requires so many carefully arranged accessories that it will not bear to be encumbered with further complications.

V. Fifthly, there is a certain caution always to be observed in comparing the results of experiment upon different species of animals, since experience shows that these results are often not the same; for nearly all kinds of animals have certain peculiarities of function which distinguish them from other species, however nearly related. They are peculiar in their mode of life, in the mechanism of their digestion or circulation, or in the excitability of certain organs or tissues. Even the constitution of the animal fluids, though presenting a general resemblance among all the quadrupeds, and even all the vertebrate animals, presents specific differences in particular instances. Thus, the urine contains, as its characteristic ingredients, urea and alkaline urates in man and the carnivorous animals, urea and alkaline hippurates in oxen and horses, and salts of uric acid, without urea, in birds and reptiles. The peculiar ingredients of the bile are so similar in all animals in which it has yet been examined, that they may be detected by the same test (Pettenkofer's test); and yet they are specifically distinct in man, in the ox, the dog, the cat, the pig, &c., and may be distinguished by special reactions.

All these peculiarities are to be borne in mind when we apply the result of one experiment to another species of animal, or the result of experiments on animals to the human species. On examining the nature of this dissimilarity and resemblance which exist at the same time between different kinds of animals, we shall see how far the resemblance between them may be trusted, and why, in certain cases, a comparison will not hold good.

I have already called your attention to the fact that physiological phenomena are complicated in their nature, and that the results of physiological experiment are liable to be complicated in the same degree. In fact, nearly every experiment or operation upon the living body has two different results, viz., a *direct* result and an *indirect* result; the direct result being due immediately to the effect of the operation upon the particular organ interested, and to nothing else; and the indirect result following, in a secondary manner, upon the disturbance caused among several contiguous organs, and to the reaction between them. Thus the direct effect of division of the spinal cord is loss of voluntary motion and sensibility in the parts below; its indirect effects are retention of the urine from paralysis of the bladder, decomposition of the urea into carbonate of ammonia, an irritating condition of the excreted fluid, and inflammation of the urinary passages. Now, it is found that in all vertebrate animals the general plan of structure and function is so simliar that the *direct* effect of an experimental operation is always the same, or varies only in rapidity, intensity, and duration. Thus in all animals the anterior roots of the spinal nerves are found to be motor in their function, and the posterior roots sensitive. In all, the lungs absorb oxygen and exhale carbonic acid. In all, an acid gastric juice is secreted by the stomach on the entrance of food, which it disintegrates and liquifies by a process of chemical solution and transformation.

We find, however, that there is often a great difference in the rapidity with which certain effects are produced. Thus, in all animals a complete stoppage of respiration produces death; but in birds and quadrupeds the fatal effect follows in a few moments, while in most reptiles death is not produced in less than from half an hour to an hour. Dogs, if kept at rest and prevented from running about, may be entirely deprived of food for eight or ten days without exhibiting any marks of suffering or debility, while rabbits require to be supplied so constantly with food that they may be starved to death in three days. In certain reptiles the capacity for abstinence is very much greater than in any warm-blooded animal. Frogs and turtles may be kept for many weeks without food, and Dr. Riddell, of New Orleans, has kept an *Amphiuma tridactylum* (aquatic lizard of the Southern waters) alive for twenty-eight months without feeding. I have myself kept a *Menobranchus lateralis* (great water-lizard of the Northern lakes) for eight months without food, at the end of which time he remained plump and vigorous; and I have been informed by Mr. Kollar, superintendent of the Department of Reptiles in the zoological museum at Vienna, that the *Proteus anguinus* has been kept in that institution for six years, without any other food than the organic matter in suspension in spring water.

There is a similar difference in the degree of sensibility to pain possessed by different animals. Some, which bear abstinence from food without difficulty, have an acute sensibility to mechanical injuries; while others, which are very delicate in regard to privations, appear to have comparatively little sensibility to pain. The action of certain drugs and poisons also varies very much in intensity in different animals. Opium exerts but little narcotic effect on dogs, even in large doses; and while strychnine produces its peculiar effect on frogs almost as readily as on the human subject, hydrocyanic acid can be made to act on these animals only with the greatest difficulty.

But the most remarkable peculiarities exhibited by experiment on different species of animals are in its *indirect* or secondary results. Paralysis of the facial nerve, for example, in the human subject produces a loss of expression in the paralyzed half of the face, and a distortion of the features to the opposite side; and if both sides be affected, there is simply a complete loss of expression, together with some difficulty in the reception and mastication of food. But in the horse, if both these nerves be divided, the effect of the operation is fatal, and the animal dies from suffocation. This is because the horse breathes exclusively through his nostrils, which are large and expansible, performing regular respiratory movements like those of the chest. When the facial nerves are divided, the muscles which expand the nostrils are paralyzed and their sides collapse; and, as the animal cannot breathe through the mouth, owing to the peculiar position of the larynx, which is on a level with the posterior nares, he necessarily dies from suffocation.

You see a very similar arrangement of the parts in this head of the sheep. The cavity of the mouth has been opened by taking away one lateral half of the lower jaw, and on looking backward toward the œsophagus, you see only the anterior surface of the epiglottis, which stands up in such a way that its edges are in contact with the lower surface of the soft palate, and the opening of the glottis is not visible from the cavity of the mouth, but communicates directly with the posterior nares; consequently, when I introduce the end of a bougie into the lower part of the trachea, and push it from below upward, it comes out, you observe, not at the mouth, but at one of the nostrils. These animals, accordingly, breathe through the nares, but not through the mouth; and if the nostrils be obstructed by paralysis of their nerves, suffocation is inevitably the result.

Again, if the Third branch of the Fifth Pair of nerves be divided on one side in the human subject, and in most animals, there is hardly any other result than a loss of sensibility in the parts about the chin and lower lip. You will remember that this nerve, beside supplying the teeth of the lower jaw and the integument about the lower part of the face, has a motor branch which supplies the muscles of mastication, or those moving the lower jaw. But after division of this nerve on one side, mastication is still performed very well by aid of the opposite muscles, and but little inconvenience results; but if the same operation be performed in the rabbit, the two gnawing teeth in front of the upper and lower jaw no longer come together in their exact position, owing to the slight lateral displacement of the lower jaw. The teeth, accordingly, which do not meet and wear each other away, as in the natural condition, grow irregularly, and produce erosions and ulcerations of the mouth; and this, together with the difficulty of using the grinding teeth in the back part of the jaw, and of properly masticating a sufficient quantity of food, produces, finally, the death of the animal, in consequence of paralysis on one side of the third branch of the fifth pair.

It is evident, therefore, that we must exercise a certain reserve in applying the results of an experiment upon the lower animals to man, or to different species of animals. If these results be direct and immediate in the mode of their production, it is always safe to make application of them to the human subject; but if they be indirect, we cannot do so with certainty until we have learned all the successive steps by which they are produced. If we find, accordingly, that the destruction of a particular organ is always followed by the death of the animal operated on, we cannot conclude from this that the organ is essential to life in the human species, or that its destruction would be fatal to other animals. For the fatal effect which follows its injury or removal may be indirectly produced by the disarrangement of other organs; and the degree or manner of its connection with these other organs may not be the same in the two kinds of animals.

VI. Lastly, it is very essential for the experimental physiologist to be acquainted always with the *individual peculiarities* of the animals upon which he operates; and for this purpose he should keep them under his observation, as a general rule, for a certain time before performing an experiment. In many cases this is necessary, simply to place the animal in a natural and healthy state of body. Dogs, for example, are usually in an unnatural condition for several days after being introduced to a new master and shut up in a strange apartment. During this time they refuse their food, digestion is suspended, the intestinal secretions stopped, and the urine either totally suppressed or retained within the bladder. The animals remain, for the most part, quiet and comparatively inexcitable, and do not exhibit their natural disposition. While in this condition, they are altogether unfit for any experiment upon the digestive or secreting organs, and the functions of the nervous system are also more or less disturbed. Usually this state of things lasts only for one, two, or three days, while, in some instances, it continues for nearly a week. In nearly all cases, however, there is more or less of it, and even where the animal appears from the first quite lively and contented, he almost invariably, during a certain time, refuses food, showing that he is not altogether in a natural condition.

At the end of two or three days, however, the animal becomes familiarized with his new quarters, and his nervous system and bodily organs return to their usual state. The intestinal secretions and the urine begin to be evacuated, and food is taken with relish. At the same time, the natural disposition of the animal is manifested, and for the first time it can be seen whether he is sluggish or excitable, quarrelsome or good tempered, voracious or abstinent. It is singular to observe how suddenly this change sometimes takes place; so that a dog that has been kept for some days and is just upon the point of getting through with this process of *acclimation*, will become so transformed in a single night as hardly to appear like the same animal.

As soon as the animal is restored to his usual condition, the observer will at once see that a certain acquaintance with his individual peculiarities is necessary, in order that the results of an experiment may be properly appreciated. These peculiarities relate not only to the irritability of the nervous system, the degree of intelligence, and the mental disposition, but they affect the entire organization, in its functions of nutrition, secretion, muscular contractibility, &c. One dog will habitually feed with great appetite and relish, devouring large quantities, and always ready for it at the usual time. Another takes his food sparingly, and is fastidious as to its quality, requiring a much smaller quantity than the first, though, to all appearances, equally well nourished. In one animal the secretions are either more abundant or more easily excited than in another, the senses more acute, and the motions more varied and vigorous. It is particularly in experiments upon the nervous system, in which the effect of an operation is indicated so much by the state of the disposition, the posture, manner of using the limbs, &c., that previous observation of the individual habits of the animal is requisite; for where the effect of an operation or experiment is to be seen in the increase or diminution of a natural function, we are unable to appreciate these changes, unless we have already a proper standard of comparison in the ordinary habits of the particular animal used for experiment.

I have thus, gentlemen, endeavored to point out the principles which should guide us in the study of physiology, and to indicate the more important practical rules which it is necessary for us to observe. In studying the phenomena of the circulation, we shall find abundant opportunity for experimental investigation. These phenomena have many elements of a physical nature. The elasticity and contraction of the bloodvessels, the mechanical subdivision and connection of different parts of the circulatory apparatus, the exudation and imbibition of fluids, and the action of hydraulic forces, all have a certain degree of influence in the movement of the circulating fluid. The chemical constitution of the blood and its changes, its variations in color and density in different regions, also take part in the same phenomena. Finally, the influence of the nervous system in increasing or diminishing the flow of blood through an organ, the effect of increased or diminished respiration, and of the activity or repose of glandular organs, all require to be successively examined in detail.

